# The Role of Inflammation in Early Left Ventricular Thrombus Formation Following ST‐Elevation Myocardial Infarction—A Matched Case‐Control Study

**DOI:** 10.1002/clc.70031

**Published:** 2024-10-16

**Authors:** Batia Litmanowicz, Moran Gvili Perelman, Michal Laufer‐Perl, Yan Topilsky, Shmuel Banai, Yacov Shacham, Shafik Khoury

**Affiliations:** ^1^ Tel Aviv University Tel Aviv Israel; ^2^ Department of Cardiology Tel Aviv Sourasky Medical Center Tel Aviv Israel

**Keywords:** inflammation post myocardial infarction, left ventricular thrombus, LVT, STEMI

## Abstract

**Background:**

There is limited data on the association between inflammation and the formation of early left ventricular thrombus (LVT) following ST‐elevation myocardial infarction (STEMI). This study aimed to explore the predictive value of several inflammatory biomarkers for LVT formation following STEMI.

**Methods and Results:**

Our cohort included 2534 consecutive patients admitted to the cardiac intensive care unit (CICU) with STEMI. The final analysis included 51 patients with LVT and 102 patients without LVT, matched for age, sex, anterior infarct and ejection fraction. Upon admission, patients with LVT had higher white blood cell counts (WBC) (12.8 ± 7 vs. 12.4 ± 4 ×10^3^/µL, *p* = 0.01), higher absolute neutrophil counts (10.5 ± 4 vs. 8.6 ± 4 ×10^3^/µL, *p* = 0.003), neutrophil‐to‐lymphocyte ratio (8.2 ± 6 vs. 4.8 ± 4, *p* = 0.04), and C‐reactive protein (CRP) levels (35.9 ± 62 vs. 18.6 ± 40 mg/L, *p* = 0.04). Peak values for WBC and CRP were also higher in the LVT group (17.8 ± 8 vs. 14.6 ± 5 ×10^3^/µL, *p* = 0.003 and 95.8 ± 82 vs. 64.2 ± 76 mg/L, *p* = 0.02, respectively). In univariate regression analysis, WBC upon admission (OR: 1.12, 95% CI: 1.02–1.21, *p* = 0.02), peak WBC (OR: 1.09, 95% CI: 1.02–1.17, *p* = 0.009), neutrophil count upon admission (OR: 1.15, 95% CI: 1.04–1.26, *p* = 0.004), and peak CRP (OR: 1.01, 95% CI: 1–1.01, *p* = 0.03) predicted LVT formation, which was also evident in multivariate regression models.

**Conclusion:**

WBC and neutrophil counts upon admission, as well as peak WBC and CRP, have additional predictive value for LVT formation following STEMI, beyond classical risk factors.

## Graphical abstract

This study aimed to explore the predictive value of several inflammatory biomarkers for left ventricular thrombus (LVT) formation following ST‐elevation myocardial infarction (STEMI). White blood cells (WBC) and neutrophil counts upon admission, and peak WBC and CRP, have additional predictive value for LVT formation following STEMI, beyond classical risk factors.

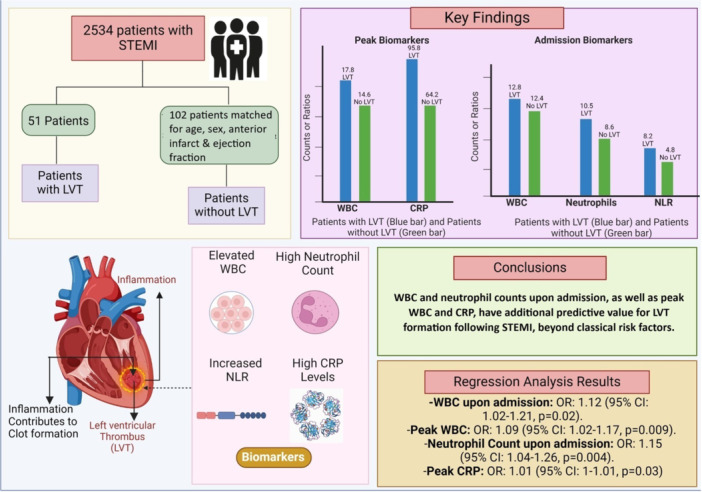

## 1 Introduction

Left ventricular thrombus (LVT) is a serious complication of ST‐elevation myocardial infarction (STEMI). Early LVT, occurring within 7 days of STEMI, is detectable in 1.5%–4% of patients using echocardiography [1, 2, 3, 4] and is found at even higher rates in patients undergoing cardiac magnetic resonance (CMR) imaging [5].

Some of the most significant traditional risk factors for early LVT formation in the era of percutaneous coronary intervention (PCI) are anterior infarct location, large infarct size, and lower left ventricular ejection fraction (LVEF) [6, 7, 8, 9]. However, the fact that only a subset of patients with these risk factors go on to develop early LVT suggests that additional mechanisms are involved. One such mechanism is the inflammatory response triggered by myocardial infarction (MI) [10]. Indeed, small studies have suggested that the relationship between stagnant flow and the formation of LVT may be influenced by a post‐MI inflammatory process [11, 12, 13]. Many of these studies, however, were hindered by variations in typical risk factors, limiting their ability to assess the additional value of inflammatory markers in this population. Understanding the effect of inflammatory processes on the risk for early LVT beyond traditional risk factors has the potential to improve risk stratification and guide more personalized monitoring and treatment.

Therefore, we conducted a matched case‐control study to mitigate the variability of typical risk factors and assess the additional role of inflammation in the formation of early LVT following STEMI.

## 2 Methods

### 2.1 Study Design and Population

**Figure 1:**
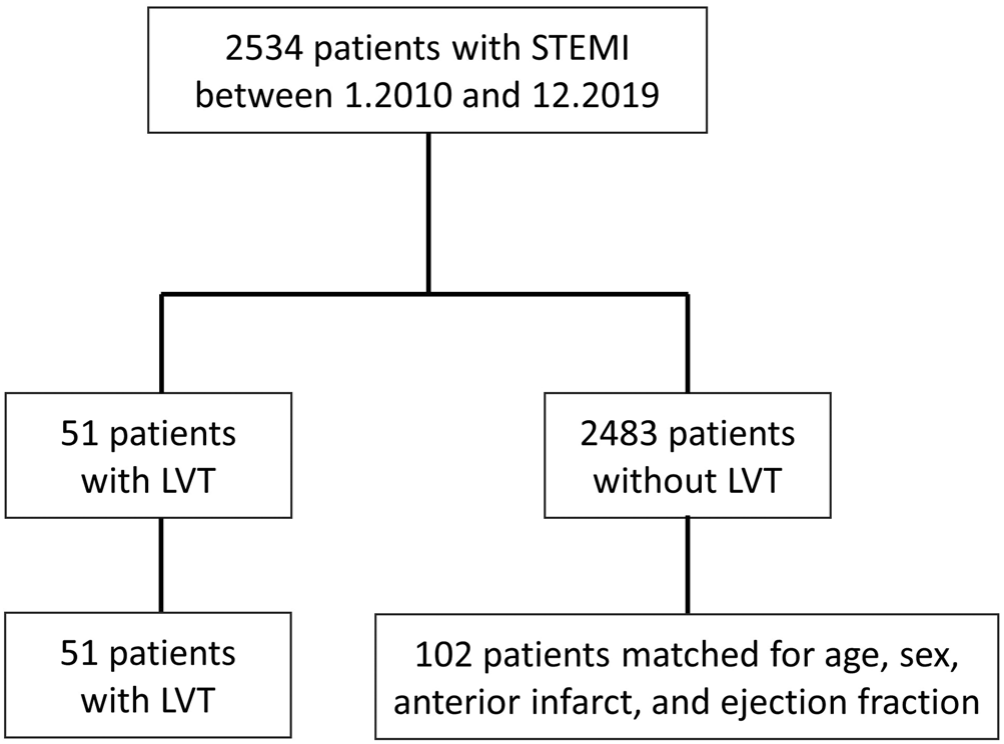
Patients' selection and matching. LVT, left ventricular thrombus; STEMI, ST‐elevation myocardial infarction.

We performed a retrospective, single‐center observational study in Tel‐Aviv Sourasky Medical Center, a tertiary referral hospital with a 24/7 PCI service. We included consecutive patients admitted to the cardiac intensive care unit (CICU) with the presumed diagnosis of STEMI between January 2010 and December 2019, all undergoing primary PCI. We excluded patients who were discharged with a diagnosis other than STEMI (e.g., stress cardiomyopathy, myocarditis), patients treated conservatively, and patients who died before a first echocardiographic test was performed. Our final cohort included 2534 consecutive patients admitted to CICU with a diagnosis of STEMI between January 2010 and December 2019. A total of 51 patients (2%) had an early LVT on echocardiography post STEMI. The control group consisted of 102 individuals matched for age, sex, anterior STEMI, and LVEF (Figure 1).

All patients received a heparin bolus (4000 U) and dual antiplatelet therapy consisting of aspirin (a loading dose of 300 mg followed by 100 mg/day) and either clopidogrel (a loading dose of 300 mg followed by 75 mg/day), prasugrel (a loading dose of 60 mg followed by 10 mg/day), or ticagrelor (a loading dose of 180 mg followed by 90 mg/day). Patients who arrived at the hospital by ambulance were administered aspirin and heparin while being transported. All patients were treated with heparin boluses during angioplasty, aiming to obtain an activated clotting time of 250–300 s in those treated with glycoprotein IIb/IIIa antagonists and an activated clotting time > 300 s in the others. Glycoprotein IIb/IIIa antagonist was administered during PCI at the discretion of the senior operator. Upon admission to CICU, all patients were started on a high‐dose statin.

The patients' baseline demographics, cardiovascular history, clinical characteristics, echocardiography, and laboratory results were obtained from the hospital's electronic medical records. Creatine phosphokinase (CPK) served as the main cardiac biomarker for this study. Troponin levels were not reported due to variations in techniques and normal reference ranges over the study period, which precluded their inclusion. The complete blood count parameters were measured using a Coulter STKS electronic counter (Coulter Corp., Miami, FL). Wide‐range C‐reactive protein (CRP) analysis was performed by the Bayer wide‐range assay. The criteria for the diagnosis of STEMI were consistent with the European Society of Cardiology/American College of Cardiology expert consensus document [14]. The study was reviewed and approved by the Institutional Review Board in accordance with the ethical standards laid down in the 1964 Declaration of Helsinki and its later amendments, with a waiver of informed consent (TLV‐0111‐18).

### 2.2 Assessment of Echocardiographic Characteristics

Echocardiography was performed using Philips iE33 (Philips Medical Systems, Andover, Massachusetts) and GE Vivid 3 (GE Healthcare, Waukesha, Wisconsin) models equipped with S5‐1 transducers. Parasternal long‐ and short‐axis, apical and 2–4 chambers views were obtained using standard transducer positions.

The 16‐segment model was used for scoring the severity of segmental wall motion abnormalities, according to the American Society of Echocardiography [15]. An LVT was defined as an echo‐dense mass adjacent to an abnormally contracting myocardial segment. It had to be distinguishable from the underlying myocardium, have a clear thrombus‐blood interface, and be visible in at least two transducer positions. If the diagnosis of LVT was suspected or could not be ruled out, serial echocardiographic tests were mandated to determine the final diagnosis.

### 2.3 Statistical Analysis

Statistical analysis was performed using IBM SPSS Statistics (Version 29.0.0.0). Continuous variables were presented as mean ± standard deviation and median with interquartile range. Categorical variables were reported as counts and percentages. Comparisons between groups were made using the Whitney–Mann test or *t* test for continuous variables and *χ* ^2^ analysis for categorical variables. Univariable and multivariable regression analyses were applied to determine predictors of LVT formation. In multivariate analysis, we adjusted for the following clinical variables irrespective of *p* value: hypertension, diabetes, smoking, and hyperlipidemia. Statistical significance was assumed at *p* < 0.05.

## 3 Results

A total of 153 patients (51 with LVT and 102 matched controls) were included in the analysis. All participants underwent PCI with a median door‐to‐balloon time of 60 min [IQR: 39–90]. The first echocardiography was performed in all patients within 1.3 ± 1.2 days. A second echocardiography study was performed during a hospital stay in 68/151 (45%) of patients (27 in the LVT group and 41 in patients without LVT) within 4.8 ± 1.6 days following admission. The decision to perform a second echocardiography was at the discretion of the treating physician, based on clinical judgment, findings on the first echocardiography and risk for LVT. For example, if the first echocardiography study was not able to confidently rule out an LVT, a second echocardiography was performed.

Among patients with LVT, a thrombus was clearly seen on the first echocardiography in 24/51 patients (47%), while in 27/51 patients (53%), a thrombus was confidently visualized only on the second echocardiography test.

There was no statistically significant difference between the LVT group and the matched‐control group in clinical characteristics, number of diseased vessels, and in left atrial and ventricular size. There was also no difference in time to reperfusion and in door‐to‐balloon time (Table 1).

**Table 1:** Clinical and echocardiographic characteristics.

Variable	All, 153	LVT, 51 (33%)	No LVT, 102 (67%)	*p* value
Age (years)	64 ± 11.5	64 ± 11.5	64 ± 11.4	0.98
Male sex	138 (90%)	46 (90%)	92 (90%)	1
Hypertension	62 (41%)	18 (35%)	47 (46%)	0.28
Diabetes	30 (20%)	6 (12%)	24 (24%)	0.08
Dyslipidaemia	78 (51%)	21 (41%)	57 (56%)	0.09
Smoking history	79 (52%)	21 (41%)	58 (57%)	0.07
Number of diseased vessels				0.36
1	65 (43%)	25	40	
2	38 (25%)	12	26	
3	44 (29%)	11	33	
Anterior infarction	132 (86%)	44 (86%)	88 (86)	1
Door to balloon (min)	60 [39–90]	59 [39–90]	60 [36–90]	0.92
Time to reperfusion (min)	267 [125–790]	290 [161–1463]	195 [113–758]	0.53
Ejection fraction (%)	38.9 ± 4.5	38.9 ± 4.5	38.9 ± 4.5	1
LA volume/BSA (mL/m^2^)	32.4 ± 10.3	31.6 ± 9.8	32.7 ± 10.5	0.64
LVEDd/BSA (mm/m^2^)	26.1 ± 3	25.5 ± 3.4	26.4 ± 2.7	0.2
LVESd/BSA (mm/m^2^)	17.6 ± 4.3	17.6 ± 3.9	17.7 ± 4.5	0.98

*Note:* Variables are *n* (%); mean ± SD; median (IQR).

Abbreviations: BSA, body surface area; LA, left atrium; LVEDd, left ventricular end‐diastolic diameter; LVESd, left ventricular end‐systolic diameter; LVT, left ventricular thrombus.

There was no difference between the groups in admission and peak levels of CPK and serum creatinine. Also, hemoglobin, glucose, and hemoglobin A1C levels did not differ significantly between the groups at presentation.

Upon admission, patients with LVT had higher white blood cell counts (WBC) (12.8 ± 7 vs. 12.4 ± 4 ×10^3^/µL, *p* = 0.01), higher absolute neutrophil counts (10.5 ± 4 vs 8.6 ± 4 ×10^3^/µL, *p* = 0.003), neutrophil‐to‐lymphocyte ratio (8.2 ± 6 vs. 4.8 ± 4, *p* = 0.04), and CRP levels (35.9 ± 62 vs. 18.6 ± 40 mg/L, *p* = 0.04). Peak values for WBC and CRP were also higher in the LVT group (17.8 ± 8 vs. 14.6 ± 5 ×10^3^/µL, *p *= 0.003 and 95.8 ± 82 vs. 64.2 ± 76 mg/L, *p* = 0.02, respectively) (Table 2).

**Table 2:** On‐admission and peak laboratory values in patients with and without left ventricular thrombus.

Variable	LVT, 51 (33%)	No LVT, 102 (67%)	*p* value
CPK units/L (admission) (U/L)	932 [259–1662]	683 [316–1752]	0.2
CPK units/L (peak), U/L)	1761 [623–2747]	1738 [789–2880]	0.91
Serum creatinine (admission) (mg/dL)	1.22 ± 0.6	1.12 ± 0.4	0.22
Serum creatinine (peak) (mg/dL)	1.46 ± 0.9	1.31 ± 0.9	0.36
Glucose (admission) (mg/dL)	166 ± 76	156 ± 60	0.38
HbA1C (admission) (%)	6.4 ± 1.8	6.3 ± 1.4	0.71
Hemoglobin (admission) (g/dL)	14.9 ± 1.5	14.8 ± 1.1	0.89
WBC (admission) (×10^3^/µL)	12.8 ± 7	12.4 ± 4	0.01
WBC (peak) (×10^3^/µL)	17.8 ± 8	14.6 ± 5	0.003
Neutrophils (admission) (×10^3^/µL)	10.5 ± 4	8.6 ± 4	0.003
Lymphocytes (admission) (×10^3^/µL)	2.9 ± 5	2.8 ± 2	0.78
NLR (admission)	8.2 ± 6	4.8 ± 4	0.04
C‐reactive protein (admission) (mg/L)	35.9 ± 62	18.6 ± 40	0.04
C‐reactive protein (peak) (mg/L)	95.8 ± 82	64.2 ± 76	0.02

*Note:* Variables are mean ± SD; median (IQR).

Abbreviations: CPK, creatine phosphokinase; HbA1C, hemoglobin A1C; LVT, left ventricular thrombus; NLR, neutrophil‐to‐lymphocyte ratio; WBC, white blood cells.

In univariate regression analysis, multiple inflammatory markers predicted the formation of early LVT. This included WBC upon admission (OR: 1.12, 95% CI: 1.02–1.21, *p *= 0.02), peak WBC (OR: 1.09, 95% CI: 1.02–1.17, *p* = 0.009), neutrophil counts upon admission (OR: 1.15, 95% CI: 1.04–1.26, *p* = 0.004), and peak CRP (OR: 1.01, 95% CI: 1–1.01, *p* = 0.03). In multivariate regression models, after adjustment for diabetes, smoking, hypertension, and hyperlipidemia, these inflammatory markers were still able to predict LVT formation (Table 3).

**Table 3:** Binary logistic regression analysis for the prediction of left ventricular thormbus.

Variable	Univariate OR (95% CI)	*p* value	Multivariate OR (95% CI)	*p* value
			*Model A*	
WBC (admission)	1.11 (1.02–1.21)	0.02	1.14 (1.03‐1.26)	0.01
			*Model B*	
WBC (peak)	1.09 (1.02–1.17)	0.009	1.1 (1.02‐1.18)	0.01
			*Model C*	
Neutrophils (admission)	1.15 (1.04–1.26)	0.004	1.18 (1.06‐1.31)	0.002
NLR (admission)	1.08 (0.99–1.17)	0.07	—	
C‐reactive protein (first)	1.01 (1–1.01)	0.06	—	
			*Model D*	
C‐reactive protein (peak)	1.01 (1–1.01)	0.03	1.01 (1–1.01)	0.04

*Note:* All multivariate models were adjusted for hypertension, diabetes, smoking and hyperlipidaemia.

Abbreviations: NLR, neutrophil‐to‐lymphocyte ratio; WBC, white blood cells.

## 4 Discussion

LVT is a serious complication of MI. Some of the most important risk factors for its occurrence are anterior infarct location, larger infarct size, and worse LVEF [4, 6, 7, 8, 9]. Nevertheless, in the era of primary PCI, only a subset of patients with these determinants will eventually develop LVT, emphasizing that more data are required to improve the detection of at‐risk populations for LVT formation following STEMI.

Previous studies have suggested that the relationship between stagnant flow and the formation of LVT may be modified by a post‐MI inflammatory process triggered by endothelial injury [11, 12, 13, 16]. However, most of these studies were limited by variations between the groups that hindered their ability to reach convincing conclusions.

In the current study, we attempted to reduce variations between the groups by matching the control group for demographics and classical risk factors for LVT. We then demonstrated that inflammatory markers, particularly WBC and neutrophil counts upon admission, as well as peak WBC and CRP levels, can predict LVT formation following STEMI.

Higher WBC and neutrophil counts have been associated with worse outcomes following MI [17]. Very few studies have suggested a trend towards higher WBC and neutrophil counts in patients with LVT, but these studies failed to show this effect in regression analysis [16]. Using a matched‐control group, we have shown for the first time that WBC and neutrophil count upon admission as well as peak WBC predict LVT formation.

The association between CRP levels and LVT formation following MI has been demonstrated in small studies [11, 12, 13, 18]. Anzai et al. showed that a peak CRP during hospitalization predicted LVT formation [12]. In those earlier reports, however, echocardiography was performed later (i.e., in the second week of hospitalization), and not all patients underwent primary PCI. Lechner et al. recently showed that peak CRP levels can predict LVT formation on CMR imaging following STEMI [16]. Indeed, CMR appears to be superior to other modalities in the detection of small LVT [5]; however, CMR studies have also shown that LVT can be present early post‐MI and self‐resolve thereafter [19], which calls into question the clinical significance of small LVT detected only by CMR. Furthermore, in clinical practice, CMR is not routinely used as a screening modality for LVT and is more likely to be utilized on a case‐by‐case basis.

Following MI, the necrotic endocardium releases several cytokines which contribute to the inflammatory and hypercoagulable state [15, 16, 17]. CRP has been shown to localize in infarcted human myocardium, and to be co‐deposited with activated complement within the area of infarct [20]. Experimental data showed that human CRP increases the size of MI by activating complement [21] and pathological analysis of infarcted human myocardium showed that CRP may function as a local pro‐inflammatory mediator during an acute MI by activating complement [22].

Thus, elevation of serum CRP levels in acute MI patients may not be an epiphenomenon of the infarction, but may rather directly contribute to local inflammation and thrombus formation. Indeed, inflammation and thrombosis appear to be closely related in several clinical setting [23, 24, 25, 26, 27]. Katayama et al. reported a relationship between inflammation and subacute stent thrombosis in patients with acute MI treated with primary coronary stenting [24], and lowering CRP levels by the use of statins in an apparently healthy population was associated with decreased future cardiovascular risk [28, 29]. Taken together, this data indicate that elevated serum CRP concentrations in STEMI patients may partially reflect an enhanced inflammatory response contributing both to coronary vulnerability and exaggerated inflammatory and hypercoagulable response from myocardial injury.

Our findings have several clinical implications. The use of inflammatory markers may have additional value in identifying patients who are at risk for developing LVT beyond traditional risk factors. Patients with classical risk factors who also exhibit elevated inflammatory markers may benefit from serial echocardiography tests before discharge or enhanced imaging modalities such as contrast echocardiography and CMR.

A strength of our study is its reflection of real‐world practice, where echocardiography is commonly used as a screening tool for LVT, while more advanced imaging techniques are reserved for cases where echocardiography results are inconclusive or the risk of LVT is considered high.

Current guidelines do not support the routine use of prophylactic anticoagulation therapy after primary PCI to prevent LVT. Instead, they recommend treatment on a patient‐by‐patient basis [30]. The 2013 ACC/AHA STEMI guidelines, based on Level of Evidence C, gave a Class IIb indication (may be considered) for prophylactic anticoagulation among patients with STEMI and anterior apical akinesis or dyskinesis at risk for LVT, with a duration that can be limited to 3 months [31]. The risk of LVT formation after MI may be greatest in the first 2 weeks [32]. In this context, our findings suggest that patients with classical risk factors who also have elevated inflammatory markers, particularly WBC and neutrophil counts upon admission, as well as peak WBC and CRP levels, may be considered for a short course of anticoagulation therapy to reduce the risk of LVT formation.

The role of inflammation in LVT formation suggests that anti‐inflammatory medications, such as colchicine, may mitigate the risk of LVT formation in a subset of patients who exhibit an enhanced inflammatory response following MI. This suggestion is informed by our current understanding of colchicine's effectiveness in reducing inflammation and cardiovascular events post MI [33]. However, further studies are needed to validate this approach.

## 5 Limitations

This is a retrospective single‐center observational study which may introduce detection and selection bias. Not all patients in the control group underwent a second echocardiography and hence some LVT may have been missed in this group. However, given the low incidence of LVT, this is not expected to have a significant impact on our study. When compared to CMR, non‐contrast echocardiography has a 35% sensitivity for the diagnosis of LVT while contrast echocardiography significantly improved the sensitivity for LVT diagnosis to 64% [34]. It is possible that some LVTs were missed in our cohort due to the routine use of non‐contrast echocardiography. To enhance the detection power and reduce the false results associated with the use of non‐contrast echocardiography, we used multiple tomographic planes, frequent adjustment of gain, depth focus, color Doppler with low scale as a contrast substitute, and a high transducer frequency. Finally, we only report early LVT detected during hospital stay while late LVT formation has been demonstrated in echocardiographic studies performed 1–3 months after discharge [35, 36].

## 6 Conclusions

WBC and neutrophil counts upon admission, as well as peak WBC and CRP levels, have additional predictive value for LVT formation following STEM, beyond classical risk factors. Further studies are required to explore the potential interventions based on these findings.

## Conflicts of Interest

The authors declare no conflicts of interest.

## Data Availability

The data that support the findings of this study are available from the corresponding author upon reasonable request.
